# Sind die Konzepte zur „chronischen Manie“ der deutschsprachigen Psychiatrie um 1900 frühe Beiträge zum Störungsbild der adulten Form der Aufmerksamkeitsdefizit‑/Hyperaktivitätsstörung (ADHS)?

**DOI:** 10.1007/s00115-024-01669-7

**Published:** 2024-05-15

**Authors:** Steffen Müller, Maria Strauß, Holger Steinberg

**Affiliations:** 1 Klinik und Poliklinik für Psychiatrie und Psychotherapie, Universitätsklinikum Leipzig AöR, Leipzig, Deutschland; 2 Forschungsstelle für die Geschichte der Psychiatrie, Klinik und Poliklinik für Psychiatrie und Psychotherapie, Medizinische Fakultät der Universität Leipzig, Semmelweisstraße 10, 04103 Leipzig, Deutschland

**Keywords:** Aufmerksamkeitsdefizit‑/Hyperaktivitätsstörung (ADHS), Erwachsene, Geschichte der Psychiatrie, Klassifikation, Krankheitsentität, Adult attention deficit hyperactivity disorder (ADHD), Adults, History of psychiatry, Classification, Illness entity

## Abstract

Die adulte Form der Aufmerksamkeitsdefizit-/Hyperaktivitätsstörung (ADHS) rückt immer mehr in den Fokus der Erwachsenenpsychiatrie. Trotz längst etablierter diagnostischer Kriterien des Störungsbilds und spezifischer Therapieansätze hört man in der heutigen Diskussion Vorurteile, dass ADHS eine „Modeerscheinung“ sei. Die Psychiatriegeschichte kann hier einen aufklärenden Beitrag leisten und versuchen aufzuzeigen, dass es sich bei der adulten Form der ADHS um ein konstant existentes Krankheitsphänomen handelt. Die vorliegende Studie arbeitet die um 1900 in der deutschsprachigen Psychiatrie geführte Diskussion z. T. prominenter Autoren über die „chronische Manie“ auf. Die einzelnen Konzepte wurden inhaltlich analysiert, miteinander und mit den heutigen Diagnosemanualen zur adulten ADHS verglichen. Das Ziel dieser Arbeit ist es, zu hinterfragen und zu diskutieren, ob diese „chronisch-manischen Konzepte“ zur Ideengeschichte der adulten ADHS gehören und ob also mit deren Hilfe eine Lücke in der Überlieferungsgeschichte dieser Störung gefüllt werden kann. Wir gelangen zu dem Schluss, dass Nervenärzte des frühen 20. Jahrhunderts über Patienten berichteten und diskutierten, die mit großer Sicherheit heute die Diagnose ADHS erhalten würden. Die Psychiater hatten Schwierigkeiten, diese Störung in ihre nosologischen Schemata einzuordnen, doch ihre „chronisch-manischen Konzepte“ lassen deutliche Parallelen zu den heute gängigen Diagnosekriterien der adulten ADHS und ihrer Symptomatik erkennen.

## Abstract (en)

The adult form of attention deficit hyperactivity disorder (ADHD) has increasingly become a focus of adult psychiatry. Despite long-established diagnostic criteria and specific therapeutic approaches for the disorder, the common misconception that ADHD is a “fad” has persisted. Examining the history of psychiatry can make an educational contribution by showing that the adult form of ADHD is a continuously existing illness phenomenon. The present study examines the discussion of sometimes prominent authors about “chronic mania” in German-speaking psychiatry around 1900. The individual concepts were analyzed for their content and compared with each other and with modern diagnostic manuals for adult ADHD. The aim of this work is to question and discuss whether these “chronic-manic concepts” are part of the conceptual history of adult ADHD and whether a gap in the history of this disorder can be filled with their help. It is concluded that in the early twentieth century neurologists reported and discussed about patients who nowadays would almost certainly receive the diagnosis of ADHD. The psychiatrists had difficulty in classifying this disorder into their nosological schemes but their “chronic-manic concepts” show clear parallels to the current diagnostic criteria for adult ADHD and its symptoms.

Das Störungsbild der adulten ADHS ist spätestens seit den 1980er-Jahren zunehmend auch in den Fokus der Erwachsenenpsychiatrie gerückt. Aufgrund des noch geringen Bekanntheitsgrads außerhalb von spezialisierten Ambulanzen wird nicht selten von einer „Modediagnose“ gesprochen. In diesem Zusammenhang stellt sich die Frage, inwieweit sich in der zeitlich weiter zurückliegenden psychiatrischen Fachliteratur Beschreibungen von ADHS-Symptomen identifizieren lassen. Zu Beginn des 20. Jahrhunderts diskutierten namhafte Nervenärzte wie Kraepelin, Wernicke, Bleuler oder Jung kontrovers über „chronisch-manische Konzepte“. Ob sich innerhalb dieses diagnostischen Konstrukts aus heutiger Sicht typische ADHS-Symptome finden lassen, soll diese Arbeit klären.

## Einleitung

Die Aufmerksamkeitsdefizit‑/Hyperaktivitätsstörung (ADHS) bei Erwachsenen steht immer mehr im Fokus des allgemeinpsychiatrischen Alltags. Man geht derzeit von einer Verbreitung von 2,5 bis 6,2 % in der Bevölkerung aus [11]. Dazu im Widerspruch steht die vollkommen mangelhafte psychiatriegeschichtliche Zuwendung zum Fragenkomplex der Existenz oder Nichtexistenz der adulten Form der ADHS.

Einen ersten Überblick zur Geschichte der ADHS lieferten beispielsweise Martinez-Badía und Martinez-Raga. Sie zeigen, dass die adulte Form des Störungsbilds, im Gegensatz zur kindlichen Form, in der psychiatriegeschichtlichen Forschung bis heute wenig beachtet wurde [19]. In ersten vorliegenden historischen Abhandlungen der adulten ADHS [3, 4] wird der Fuldaer Arzt Melchior Adam Weikard (1742–1803) als Erstbeschreiber erwähnt, welcher im Jahr 1775 in einer anonym veröffentlichten Schrift [2] über charakteristische Symptome der ADHS bei Erwachsenen berichtete. Auch der Schotte Alexander Crichton (1763–1856) beschrieb der Kurzübersicht von Martinez-Badía und Martinez-Raga folgend [19] im Jahr 1798 typische Symptome der adulten ADHS. Als wegweisend für das Verständnis und die Konzeptualisierung der kindlichen ADHS gilt der Londoner Kinderarzt George Still (1868–1941), der das Störungsbild erstmalig 1902 einem größeren medizinischen Fachpublikum zugänglich machte [29]. In den darauffolgenden Jahren berichteten die beiden Berliner Psychiater Franz Kramer (1878–1967) und Hans Pollnow (1902–1943) über eine „hyperkinetische Erkrankung im Kindesalter“, welche aus heutiger Sicht unter dem Namen des „Kramer-Pollnow-Syndroms“ einen essenziellen Bezugspunkt für die frühe Konzeptualisierung der ADHS im deutschsprachigen Raum darstellt [21].

Angesichts dieser frühen Beschreibungen verstetigt sich der Eindruck, dass die adulte Form der ADHS keine neue Erscheinung der letzten Jahrzehnte darstellt, gleichwohl sie auch erst seit 1980 als diagnostische Gruppe im Diagnostic and Statistical Manual of Mental Disorders (DSM) III als „Attention Deficit Disorder Residual Type“ (ADD-RT) aufgeführt wurde [1]. Es stellt sich daher die Frage, ob frühere Nervenärzte entweder diesen Patiententypus nicht erkannt haben, auch weil sie ihm zu selten begegneten (s. die Auffassung einer derzeitigen Modediagnose), oder ihn alternativ in anderen, bereits bestehenden Entitäten klassifizierten.

Erst kürzlich konnte herausgearbeitet werden, dass Emil Kraepelin (1856–1926) kurz nach 1900 in seinem Lehrbuch für Psychiatrie Patienten beschrieben hat, welche nach heutigen Kriterien die Diagnose einer adulten ADHS erhalten würden [27]. Kraepelin ordnete u. a. in einer Art „Grundzustand“ des „manisch-depressiven Irreseins“, welchen er als „konstitutionelle Erregung“ oder „manische Veranlagung“ betitelte, Patienten ein, welche aus heutiger Sicht durch ADHS-spezifische Symptome auffielen. Seinen Literaturverweisen folgend konnten in einer weiteren Arbeit [28] andere Nervenärzte identifiziert werden, die kurz zuvor in einzelnen Fallbeschreibungen innerhalb ihrer „chronisch-manischen Konzepte“ ebenfalls Betroffene vorstellten, auf die nach heutigen Gesichtspunkten die wesentlichen Kernsymptome der adulten ADHS zutreffen.

## Methoden

Diese Arbeit folgte dem aufgezeigten Pfad der „chronisch-manischen Konzepte“. Dabei wurde sichtbar, dass zu diesem nosologischen Konstrukt in der deutschsprachigen Psychiatrie um und vor allem kurz nach 1900 mehrere, z. T. prominente Autoren Beiträge publizierten. Ausgangspunkt der Literaturrecherche waren Kraepelins Schilderungen zur „konstitutionellen Erregung“ des „manisch-depressiven Irreseins“ [17, 27]. Seinen und den nachfolgenden Literaturhinweisen folgend, konnten somit weitere historische Beiträge identifiziert werden. Ferner wurde für die Erfassung zusätzlicher potenzieller Quellen das damals weitverbreitetste deutschsprachige, klinisch-anwendungspraktisch orientierte psychiatrische Fachblatt, die „Allgemeine Zeitschrift für Psychiatrie und psychisch-gerichtliche Medizin“, chronologisch und systematisch nach Veröffentlichungen zu diesem Themenbereich im Zeitraum um 1900 durchsucht. Die hierbei ermittelten, relevantesten Fallberichte und Kapitel aus Lehrbüchern der Psychiatrie dienten der Studie als Primärquellen. Die einzelnen Texte wurden inhaltlich analysiert und miteinander sowie mit den heutigen Diagnosemanualen zur adulten ADHS verglichen. Hierbei galt es unter anderem, die jeweiligen unterschiedlichen Entwicklungstrajektorien, aber auch Parallelen zu erfassen.

## Ergebnisse

Als zeitlicher Ausgangspunkt dieser Untersuchung wird die 1881 veröffentlichte und sich in der Folge als Standardwerk zur Manie erweisende Monografie des in Berlin tätigen Nervenarztes *Emanuel Mendel* (1839–1907) gesetzt [20]. Mendel lehnt darin den Begriff der chronischen Manie vollständig ab. Er sieht in den Beschreibungen der chronischen Manie insbesondere sekundäre psychopathische Zustände geistiger Schwäche mit einhergehenden Erregungszuständen und keine eigene Krankheitsentität [20, S. 95–97]. So meint er, „… erscheint mir die Unterscheidung zwischen der acuten und der chronischen Form der Manie weder praktisch wichtig, noch durch die Thatsachen gefordert, und es erscheint mir zweckmässiger, den Namen ‚chronische Manie‘ ganz aufzugeben“ [20, S. 96–97]. Der prominente Nervenarzt und Sexualwissenschaftler *Richard von Krafft-Ebing* (1840–1902) unterscheidet in seinem Lehrbuch der Psychiatrie wenige Jahre später wiederum eine akute von einer chronischen Form der Tobsucht. Letztere definiert er als Manie mit einem prolongierten Verlauf und einer „Gesamtdauer von Monaten bis über [die] Jahresfrist“. Diese sei „meist eingeleitet durch ein melancholisches Prodromalstadium“ [31, S. 376]. Auch *Theodor Ziehen* (1862–1950) betont 1902, dass die chronische Form der Manie, welche unverändert bis zum Lebensende anhalte, grundsätzlich zwar existiere, jedoch eine Seltenheit sei. Er definiert diese als eine Form, in welcher die Symptome der heiteren Stimmung, der Ideenflucht und des Bewegungsdrangs ohne Intelligenzstörungen viele Jahre anhalten könnten. Grundsätzlich könne diese Spielart jedoch „noch in Heilung übergehen“ [33, S. 332–333].

Ein anderes, präziseres Bild der chronischen Manie präsentiert *Carl Wernicke* (1848–1905) und leitet damit einen ersten Paradigmenwechsel ein. In seinem „Grundriss der Psychiatrie“ aus dem Jahr 1900 beschreibt der Breslauer Ordinarius neben der akuten Manie die für ihn als „eigenes Krankheitsbild“ [32, S. 369] existierende Form der chronischen Manie. Er betont gleich zu Beginn seiner Ausführung, dass eine akute Manie niemals in eine chronische Manie übergehe und grenzt sich hiermit klar von anderen Konzepten seiner Zeit ab. So weise die chronische Manie „alle wesentlichen Kennzeichen der acuten Manie [auf], [jedoch] nur so modificirt, wie es die Bedingungen eines chronischen, stabilen Zustandes mit sich bringen. Die Ideenflucht hält sich demgemäss in mässigen Grenzen und steht noch unter dem Einfluss einer gewissen Besonnenheit und Selbstbeherrschung. Demgemäss ist die heitere Verstimmung wenig ausgeprägt, kommt aber doch gelegentlich zum Durchbruch“. Die Erkrankten würden kontinuierlich durch Missachtung von Gesetzen und gesellschaftlichen Regeln in Probleme und Konflikte geraten [32, S. 369–370].

Der Hallenser Psychiater *Ernst Siefert* (1874–1940) beschreibt in seinem 1902 veröffentlichten Artikel „Über chronische Manie“ [25] den Fall eines 36-jährigen Arbeiters mit „eine[m] leichten Grad manischer Erregung …“, welcher durch eine „ungewöhnlich lange Verlaufsphase“ gekennzeichnet sei. So sei „sein ganzes Leben, soweit es sich überhaupt zurückverfolgen lässt, eine ununterbrochene Kette von Handlungen, die den Stempel des manisch Bedingten an sich tragen und überall die gleiche krankhafte Persönlichkeit erkennen lassen …“. Genaue Aussagen zur Häufigkeit der chronischen Manie kann Siefert keine machen. Die seltene Notwendigkeit psychiatrischer Intervention bei derartigen Erkrankten führe jedoch dazu, dass sie womöglich unterdiagnostiziert sei. Er vermute, dass „… unter Vagabonden, den Insassen von Arbeitshäusern und Gefängnissen derartige psychotische Individualitäten …“ öfters zu finden seien. Ebenso spricht Siefert sich dahingehend aus, dass ein häufig komorbid vorliegender Alkoholmissbrauch „lediglich … als eine Begleit- und Folgeerscheinung“ aufzufassen und „keinesfalls aber etwa als die Ursache der Erkrankung“ zu sehen sei [25, S. 269].

Der psychiatrische Oberarzt der königlichen Heil- und Pflegeanstalt Zwiefalten *Adolf Schott *(1874–nach 1934) stellt in seinem 1904 veröffentlichten, etwa 20-seitigen Zeitschriftenaufsatz der „Klinische Beitrag zur Lehre von der chronischen Manie“ ein ähnliches Konzept vor [24]. Er stellt heraus, dass es zwischen seinen Kollegen Meinungsverschiedenheiten zur Einordnung des Krankheitsbilds der chronischen Manie gebe. So sei es seiner Meinung nach strittig, ob es sich bei der chronischen Manie um ein eigenes Krankheitsbild handele oder ob sie den sekundären Demenzformen zuzuschreiben sei. Zu ihrer pathognomonischen Symptomatik äußert sich Schott wie folgt: „Sie ist charakterisiert durch den manischen Symptomkomplex in milderer Form als bei der akuten Manie und gekennzeichnet durch eine verhältnismäßig geringe Schädigung der Merkfähigkeit, des Gedächtnisses, der Schulkenntnisse und der geistigen Regsamkeit, während eine ethische Degeneration, sowie eine Urteilsschwäche und Kritiklosigkeit, vorherrschend in Bezug auf die eigene Person und Leistungsfähigkeit, ziemlich erhebliche Grade erreichen können“ [24, S. 19].

Auch der Züricher Psychiater und Begründer der analytischen Psychologie *Carl Gustav Jung *(1875–1961) sieht in seiner 1904 veröffentlichten Abhandlung über die „manische Verstimmung“ [15] ein Krankheitsbild, welches durch einen „meist bis in die Jugend zurückreichenden, stabilen, submanischen Symptomenkomplex“ [15, S. 39] gekennzeichnet sei. Dieses rechnet er dem Gebiet der psychopathischen Minderwertigkeit zu. Jung hält fest, dass er den Ausdruck der „chronischen Manie“, wie Wernicke und Siefert ihn definieren, für zu „stark halte“ und den Begriff der „konstitutionelle[n] manische[n] Verstimmung“ [15, S. 16–18] präferiere. Für eine konkrete Diagnosestellung müsse man sich nach Jung weiterhin an die Kardinalsymptome der Manie halten, die er in „Gemütslabilität mit vorwiegend heiterer Verstimmung, Ideenflucht, Ablenkbarkeit, Vielgeschäftigkeit (oder Bewegungsdrang)“ sieht [15, S. 17]. Ähnlich wie Siefert hält Jung fest, dass „Alkoholismus, Kriminalität, Moral insanity, soziale Unbeständigkeit oder Unfähigkeit … in diesem Falle vom submanischen Zustande abhängige Symptome“ [15, S. 17] seien. Jung versucht, seine Beobachtungen anhand einzelner Patientenfälle für seine Leser besser verständlich zu machen. Einer der insgesamt vier Fälle präsentiert einen 29-jährigen Kaufmann. Dieser sei bereits in der Schule „zerstreut und unaufmerksam“ gewesen, habe „immer Allotria“ getrieben, obwohl er sich oftmals „begabt, aber ohne Ausdauer“ präsentiert habe. Auch später im Beruf habe er sich „durch alle möglichen Vergnügungen von der Arbeit ablenken“ lassen, „war immer sehr lebhaft“ und „konnte sich zu keiner Arbeit konzentrieren“, während ihn „eine furchtbare innere Unruhe“ beständig gequält habe. Auf dieser Grundlage seien mehrfach Konflikte im beruflichen und privaten Umfeld entstanden [15, S. 18–21].

Der spätere Direktor der Heil- und Pflegeanstalten Pirna-Sonnenstein und Leipzig-Dösen *Hermann Paul Nitsche* (1876–1948) postuliert ebenfalls Fälle von „manischer Erregung“. In seinem Beitrag „Über chronisch-manische Zustände“ von 1910 fasst Nitsche zusammen, dass „eine einheitliche, allen Fällen gerecht werdende Schilderung vom Symptombilde der chronischen Manie“ anhand zuvor beschriebener Patientenfälle nicht möglich sei [22, S. 124–125]. Dabei gebe es jedoch allgemein Merkmale, welche vermehrt bei den chronisch verlaufenden Formen manischer Zustände auftreten würden, wie u. a. eine erhöhte Ablenkbarkeit, eine Weitschweifigkeit und Erinnerungsfälschungen. Zusätzlich spiele die „erbliche Belastung“ bei diesen Erkrankten eine Rolle [22, S. 126]. Obwohl gerade die „leichten Fälle, offenbar verhältnismäßig selten in die Anstalten“ kämen, also seiner Auffassung nach einer psychiatrischen Versorgung bedürften, ist sich Nitsche sicher, dass diese Fälle der konstitutionellen Erregung „recht häufig“ seien [22, S. 126]. Nitsche wurde nach dem Zweiten Weltkrieg aufgrund seiner federführenden Beteiligung an der „Aktion T‑4“ im Rahmen der NS-„Euthanasie“ und dem hundertausendfachen Mord an Patienten zum Tode verurteilt [8].

Auch in den Arbeiten *Gustav Spechts* (1860–1940), dem psychiatrischen Lehrstuhlinhaber der Friedrich-Alexander-Universität Erlangen und ehemaligen Kollegen Kraepelins, finden sich umfassende Beschreibungen zur chronischen Manie. In seinem im Jahr 1905 veröffentlichten Aufsatz über die „Chronische Manie und Paranoia“ [26] beschreibt er diese als „völlig selbstständige Geisteskrankheit“, welche er als Form der konstitutionell psychopathischen Zustände einordne [26, S. 591]. Sie imponiere durch den „manischen Erscheinungskomplex“, jedoch auf der Ebene der leichten Intensität einer Hypomanie. Leichte Exazerbationen seien seiner Meinung nach jedoch möglich. Gemäß seiner Auffassung trete der Beginn der chronischen Manie mit der „Ausreifung der Persönlichkeit“ auf. Specht hebt weiterhin hervor, dass auch die Eltern der Erkrankten oftmals psychiatrisch auffällig seien, und stellt somit in den Raum, dass die Veranlagung zur Erkrankung womöglich vererbt wird. Ein weiterer Aspekt, den Specht anspricht, ist die Einordnung der Betroffenen innerhalb eines Kontinuums zwischen gesund und krank. Einige Fälle seien seiner Auffassung nach als Grenzfälle einzuordnen, was oftmals zu Fehldiagnosen und Unterordnungen zu anderen Erkrankungen wie dem „chronischen Alkoholismus“ oder dem „epileptische[n] Irresein“ führe [26, S. 591].

Auch *Emil Kraepelin* (1856–1926) beteiligt sich an der laufenden Manikerdiskussion. Innerhalb seiner Lehrbuchbeschreibung des manisch-depressiven Irreseins, welches heute der großen Gruppe der affektiven Störungen zuzuordnen ist, geht er ergänzend auf spezielle „Grundzustände“ dieser Entität ein ([17], Bd. 3, S. 1312–1317). Er bezieht sich hierbei ausdrücklich auf seine Kollegen Specht und Nitsche, welche zuvor bereits Ähnliches beschrieben haben. Einen dieser Grundzustände des manisch-depressiven Irreseins betitelt Kraepelin als „manische Veranlagung“ bzw. „konstitutionelle Erregung“. Diese Personen würden nach seinen Beobachtungen keine Ausdauer beim Lernen zeigen, seien schnell ablenkbar und durch eine Unstetigkeit und Rastlosigkeit in ihrem Benehmen und Handeln auffallend. In ihren Entschlüssen seien sie sprunghaft und unberechenbar, sodass „ihr Leben regelmäßig eine Kette von unbesonnenen, abenteuerlichen, nicht selten auch unsinnigen und bedenklichen Handlungen“ darstelle ([17], Bd. 3, S. 1313). Bereits in der Schulzeit würden die Betroffenen durch Ungehorsam, häufiges Schwänzen und eine Abneigung gegen gründliches und ausdauerndes Lernen auffallen. Eine unstete Schullaufbahn und ein rastloser beruflicher Werdegang seien die Folge ([17], Bd. 3, S. 1313–1314). In Abgrenzung zu manischen Zuständen der manisch-depressiven Erkrankung arbeitet Kraepelin heraus, dass „Berührungspunkte dieses Krankheitsbildes mit leicht hypomanischen Zuständen“ unverkennbar seien. „Nur ist die Erregung hier noch schwächer angedeutet, und sie verläuft nicht in abgegrenzten Anfällen, sondern sie ist eine dauernde persönliche Eigentümlichkeit“ ([17], Bd. 3, S. 1316). Kraepelin ergänzt: „Allerdings scheint sich das klinische Bild oft erst in den Entwicklungsjahren deutlicher herauszubilden, unter Umständen in Form eines Umschlagens aus einer mehr depressiv gefärbten Jugendzeit“ ([17], Bd. 3, S. 1316). Er sieht in der manischen Veranlagung bzw. der konstitutionellen Erregung des manisch-depressiven Irreseins ein Störungsbild, das sich auf dem Kontinuum zwischen krank und gesund bewege, bereits im Kindesalter seinen Ursprung habe und durch eine dauerhaft bestehende persönliche Eigentümlichkeit gekennzeichnet sei.

In Anlehnung an Kraepelin greift der Züricher Psychiater *Eugen Bleuler* (1857–1939) in seinem Lehrbuch das Konzept der „konstitutionelle[n] Erregung oder manische[n] Veranlagung“ auf [7, S. 362–363]. Auch er ist der Auffassung, dass es bei einigen Patienten gewisse „Über- und Unterstimmungen“ gebe, „die einerseits andauern, anderseits nicht die Höhe einer krankhaften Verstimmung erreichen“. Dies führe mitunter dazu, dass diese „trotz ihrer Häufigkeit nur selten in psychiatrische Beobachtung“ geraten würden [7, S. 362]. Zu dieser Form der „andauernden Stimmungsverschiebung“ hält Bleuler fest: „Das manische Temperament dieser Leute disponiert zu übereilten Handlungen und zu leichtsinniger Lebensweise überhaupt, wenn es nicht durch einen besonders kräftigen Verstand und eine besonders gute Moral gezügelt wird. Deshalb finden wir hier auf der einen Seite protzige, rücksichtslose, zu Zanken und Querulieren geneigte Tunichtsgute, die keinen Nachhalt in ihren Unternehmungen haben, anderseits aber ‚sonnige Naturen‘ und große, ja genial angelegte, nicht selten künstlerisch begabte Leute von unermüdlicher Unternehmenslust“ [7, S. 362–363]. Dass Bleuler in seinen weiteren Ausführungen zu diagnostisch-ätiologischen Unklarheiten des manisch-depressiven Irreseins hinzufügt, dass scheinbar noch weitere ursächliche Störungsbilder existierten, welche jedoch noch nicht genau abgrenzbar seien, unterstreicht erneut den Diskurs seiner Zeit. So stellt er fest: „… es gibt sicher Verstimmungen anderen Ursprungs, die wir als solche noch nicht von den manisch-depressiven unterscheiden können. Wir sind deshalb in bezug auf die Abgrenzung nicht ganz sicher, wo eine dieser anderen Ursachen mitwirken könnte, und andererseits muß uns diese Tatsache vorsichtig machen, bloß aus der Veranlagung heraus die Anfälle erklären zu wollen“ [7, S. 363].

## Diskussion

Unter den „chronisch-manischen Konzepten“ findet man in der psychiatrischen Literatur zu Beginn des 20. Jahrhunderts sehr unterschiedliche Vorstellungen beschrieben. Während bei einigen Autoren, wie Siefert es treffend formuliert, die Existenz der chronischen Manie als Krankheitsbild „eine zur Zeit noch offene Frage“ sei [25, S. 261], zeigen sich Kollegen in ihrer Meinung bereits deutlich sicherer und sehen wie Wernicke in den chronisch-manischen Zuständen ein klar begrenztes „eigenes Krankheitsbild“ [32, S. 369]. Auffallend ist die häufige Zuordnung der Erkrankten in das damals noch sehr weitgefasste „Sammelbecken“ der Psychopathien. So ordnet auch Jung exemplarisch die „manische Verstimmung“ in das Gebiet der „psychopathischen Minderwertigkeiten“ ein [15, S. 39], sieht diese somit als eine Spielart der Psychopathie. Dieses maßgeblich durch den deutschen Psychiater Julius Koch (1841–1908) zum Ende des 19. Jahrhunderts entstehende Modell war während der Zeit der chronisch-manischen Konzeptdiskussion jedoch noch keineswegs gefestigt, abschließend definiert, geschweige denn in der psychiatrischen Fachwelt allgemeingültig akzeptiert [16, 23]. Der parallel herrschende „Psychopathiediskurs“ und die damit verbundene Frage nach den Grenzen zwischen dem Normalen und Krankhaften scheinen jedoch einen großen Einfluss auf die Überlegungen der Protagonisten gehabt zu haben.

Grundsätzlich wirkt es so, dass auch unter der Ärzteschaft im früheren 20. Jahrhundert Unsicherheiten bezüglich der Relevanz von Krankheitsphänomenen existierten. Trotz etablierter diagnostischer Kriterien der adulten ADHS hört man in der heutigen Diskussion oftmals weitläufig Vorurteile, welche im Kern postulieren, dass ADHS eine „Modeerscheinung“ sei. Auch in der Zeit um 1900 zeigten sich spürbar deckungsgleiche Argumentationsketten. So hält Schott fest, dass der Begriff der beschriebenen „chronisch-manischen Konzepte“ ein „noch ... wenig umgrenzter und von vielen angezweifelter“ sei [24, S. 1].

Trotz ihrer Unstimmigkeiten ordnen die Autoren ihre Beobachtungen oftmals in das große Feld der zu dieser Zeit weiter als heute gefassten affektiven Erkrankungen bzw. des „manisch-depressiven-Irreseins“ ein. Die Mehrzahl wiederum sieht in ihnen eine Spielart der Psychopathien. Es scheint jedoch so, als ob den Debattierenden trotz dieser Divergenzen die Eigentümlichkeit der Patienten klar bewusst gewesen sei. Gerade in den Beschreibungen Kraepelins, Wernickes, Jungs, Sieferts, Nitsches und Spechts zeichnet sich ein Krankheitsbild ab, welches sich im Kontinuum zwischen „gesund und krank“ bewegt, sich durch eine jahre- bis lebenslange Beständigkeit der Symptomatik und in Abgrenzung zur derzeitigen Auffassung der bipolaren Störung durch einen nicht phasenhaften Verlauf ([17], Bd. 3, S. 1316) charakterisiert. Auch wenn die einzelnen Autoren in ihren Begrifflichkeiten unterschiedliche Termini finden (chronische Manie: Wernicke, Siefert, Specht; konstitutionelle manische Verstimmung: Jung; manische Veranlagung bzw. konstitutionelle Erregung: Kraepelin, Bleuler), zeigen sich in der Gruppe der „chronisch-manischen Konzepte“ dieser Zeit deutliche Parallelen zur heutig als typisch bezeichneten ADHS-Psychopathologie (Tab. 1).

**Tab. 1:** Analogien zu den heutigen adulten ADHS-Kernsymptomen in exemplarischen Fallbeschreibungen um 1900 von Siefert, Jung, Specht, Nitsche und Kraepelin

Autor	Aufmerksamkeitsstörung	Motorische Überaktivität	Impulsivität	Krankheitsbeginn in der Kindheit
Siefert (1902), [25]	„Er war unfähig, einen Gedanken ruhig zu entwickeln, schweifte bald ab, achtete kaum noch auf Zwischenbemerkungen, überstürzte sich, kam vom Hundertsten ins Tausendste …“ (S. 365)	„mässiger Bewegungsdrang“ (S. 362)	„unstetes Wanderleben“ (S. 362) „In seinem Gebahren trug er eine eigenthümliche Mischung von gravitätischer Wichtigthuerei, komisch wirkender Eilfertigkeit und devoter Vertraulichkeit zur Schau“ (S. 363)	„… ist sein ganzes Leben, soweit es sich überhaupt zurückverfolgen lässt, eine ununterbrochene Kette von Handlungen, die den Stempel des manisch Bedingten an sich tragen und überall die gleiche krankhafte Persönlichkeit erkennen lassen …“ (S. 368)
Jung (1904), [15]	„In der Schule nach seiner Angabe zerstreut und unaufmerksam, trieb immer Allotria“; „Später in der Schule faul und oberflächlich, aber wenn er sich ernstlich Mühe gab, von großer Leistungsfähigkeit“; „war überhaupt begabt, aber ohne Ausdauer“; „ließ sich durch alle möglichen Vergnügungen von der Arbeit ablenken“ (S. 18)	„Er konnte sich zu gar keiner Arbeit konzentrieren, ‚eine furchtbare innere Unruhe‘ quälte ihn beständig, ein ‚rastloser ewiger Drang wegzukommen‘“ (S. 19)	„Man kann Patientin wegen ihres expansiven Wesens nicht zu Hause haben, sie unternimmt jeden Augenblick etwas anderes …“ (S. 27) „… oft war er auch so gereizt, daß, wenn ihn die Mutter oder Schwester etwas fragte, er an sich halten mußte, um nicht „mit beiden Fäusten auf den Tisch zu schlagen“ (S. 19)	„meist bis in die Jugend zurück reichenden, stabilen, submanischen Symptomenkomplex“ (S. 39)
Specht (1905), [26]	„… durch die Ruhelosigkeit und Sprunghaftigkeit im Denken …“ (S. 593)	„… in einer weniger intensiven Ausprägung der manischen Kardinalsymptome …“ (S. 594)	„… expansive Stimmungsrichtung …“ (S. 594) „… aus der Leidenschaft geborenen Urteilsentgleisungen …“ (S. 595)	„Die Entstehung des psychopathologischen Zustandes wird wohl immer mit der Ausreifung der Persönlichkeit zusammenfallen …“ (S. 591)
Nitsche (1910), [22]	„in der Schule durch Zerstreutheit“ auffällig gewesen; „… mangelhafter Fleiß und die Ungleichmäßigkeit seiner Leistungen …“ (S. 82)	„… dauernde leichte motorische Erregung …“; „… er gestikulierte beim Reden lebhaft, war in beständiger Unruhe, rutschte auf dem Stuhle hin und hier …“ (S. 85)	„… sei reizbar und jähzornig …“; „… leichtsinnig … unbedenklich in der Wahl seines Umgangs …“ (S. 82)	„Im ganzen gewinnen wir also bei einem Überblicke über den Lebensgang des Patienten den Eindruck, daß seine dauernde krankhafte Eigentümlichkeit sich im Keime schon in seinen Knaben- und Jünglingsjahren gezeigt … hat“ (S. 81–82)
Kraepelin (1913), ([17], Bd. 3)	„In der Regel nur geringe und namentlich sehr lückenhafte Kenntnisse, weil sie gar keine Ausdauer beim Lernen zeigen“; „ungemein leicht ablenkbar“ (S. 1312)	„Im Benehmen und Handeln tritt vor allem eine gewisse Unstetigkeit und Rastlosigkeit hervor“; „sehnen sich aber rasch wieder nach Veränderung und Abwechslung“ (S. 1313)	„vielfach auffallende und absonderliche Handlungen“; „gegen andere hochfahrend, rechthaberisch, reizbar, patzig, trotzig“ (S. 1312) „In ihren Entschlüssen sind die Kranken sprunghaft und unberechenbar. Infolgedessen ist ihr Leben regelmäßig eine Kette von unbesonnenen, abenteuerlichen, nicht selten auch unsinnigen und bedenklichen Handlungen“ (S. 1313)	In der Kindheit: „unbotmäßig, liederlich, Rädelsführer bei allen Ungehörigkeiten“; Schule schwänzen; häufige Schulwechsel, Scheitern bei Prüfungen wegen Abneigung gegen Lernen (S. 1313–1314)

Auch heute werden ADHS-typische Symptome oftmals anderen psychiatrischen Störungsbildern zugeordnet. Dies mag zum einen daran liegen, dass die Kernsymptome oftmals auch bei anderen Störungsbildern wie z. B. bei den affektiven Störungen auftreten, zum anderen aber auch daran, dass eine ADHS selten isoliert auftritt und bei einem Großteil der Betroffenen eine zusätzliche komorbide psychiatrische Erkrankung vorliegt [14]. Auffallend häufig registrieren die Debattenführer, dass Betroffene oftmals durch Substanzabhängigkeiten, insbesondere durch Alkoholkonsum, auffallen würden. Dies deckt sich klar mit der heutigen Datenlage. ADHS-Betroffene zeigen eine erhöhte Rate an Abhängigkeitserkrankungen [13, 14]. Bemerkenswert scheint in diesem Zusammenhang die Ergänzung Sieferts, welcher die Kausalkette auch aus heutiger Sicht treffend erfasst: „Als zweifellos darf wohl bezeichnet werden, dass der Alkoholmissbrauch lediglich als eine Begleit- und Folgeerscheinung des Zustandes aufzufassen ist, keinesfalls aber etwa als die Ursache der Erkrankung“ [25, S. 269].

Ein weiteres Argument, weshalb unter dem Konzept der „chronisch-manischen Zustände“ wahrscheinlich ADHS-Patienten eingeordnet wurden, ist die Tatsache, dass die damaligen Autoren klar erkannten, dass das Störungsbild bereits im Kindesalter seinen Ursprung hat. Dieses Kriterium ist auch heute noch ein wesentliches für die Diagnosestellung bei Erwachsenen [30]. So äußert Specht in diesem Zusammenhang, dass der Beginn der Erkrankung „mit der Ausreifung der Persönlichkeit“ zusammenfalle [26, S. 591], was sich mit einem Beginn in der Kindes- und Jugendzeit deckt. Auch Jung spricht exemplarisch in seinen Ausführungen von einem „meist bis in die Jugend zurück reichenden, stabilen, submanischen Symptomenkomplex“ [15, S. 39]. Ebenfalls erkennen mehrere Autoren das familiär gehäufte Auftreten bei Betroffenen. Schott spricht in diesem Zusammenhang von einer „schweren erblichen Belastung“ [24, S. 19], Nitsche von einer „erbliche[n] Belastung“ [22, S. 126]. Dies deckt sich klar mit den heute bekannten genetischen Hintergründen und der damit verbundenen familiären Prädisposition der Erkrankung [10].

Im Kontext der täglichen Anforderungen durch Schule, Beruf und des gesellschaftlichen Lebens scheint es, als ob „chronisch-manisch“ Betroffene, ähnlich wie heutzutage ADHS-Betroffene, durch ihre Symptomatik aneckten und hierdurch einen hohen Leidensdruck verspürten. So merkt Wernicke an, dass Betroffene „… sich selbst fortwährend allerlei Schwierigkeiten und Collisionen durch die Nichtachtung aller derjenigen Normen und Rücksichten, welche ihnen durch Sitte und Gesetz auferlegt werden“ [32, S. 370], schaffen würden. Auch heutzutage zeigen erwachsene ADHS-Betroffene ein erhöhtes Risikoverhalten in unterschiedlichen Situationen, übertreten häufiger Gesetze [18], weisen eher einen niedrigen sozioökonomischen Status und einen niedrigen Ausbildungsstand auf [9].

Weiterhin sind Vorurteile gegen und Stigmatisierung von ADHS-Betroffenen ein beständiges Thema [6]. Dies mag daran liegen, dass einerseits die Symptome der Betroffenen oftmals negativ attribuiert werden, andererseits ihr Leidensdruck nicht erkannt wird. Unter- und Fehldiagnosen sind die Folgen [12], eine spezifische Behandlung bleibt aus. Auch Siefert beschreibt ähnliche Erfahrungen der Betroffenen und unterstreicht hierdurch eine weitere Parallele bzw. die Kontinuität des Diskurses: „Derartige Kranke dürften dann meist als freche, durch den Trunk degenerierte Individuen aufgefasst werden, ihre Unverbesserlichkeit wäre Verkommenheit, ihre Euphorie ein nicht zu bändigender Leichtsinn, ihre Neigung zu Excessen in baccho et venere sittliche Verrohung, ihre motorischen Ueberproductionen schamlose Dreistigkeit“ [25, S. 269]. Zusätzlich würden Betroffene laut Nitsche intermittierend an einer labilen Affektlage leiden [22, S. 125–126] bzw. würden sie nach Jung „Exazerbationen von unsicherer Periodizität“ aufweisen und oftmals emotional labil sein [15, S. 39]. Diese Umschreibung ähnelt stark der heute beschriebenen ADHS-assoziierten emotionalen Dysregulation [5] und stützt damit die Annahme, dass unter den Beschreibungen der „chronisch Manischen“ mit großer Wahrscheinlichkeit ADHS-Patienten aufgeführt wurden.

## Fazit

Die deutsche Psychiatrie prägende Vertreter im frühen 20. Jahrhundert kannten und berichten über erwachsene Patienten mit einer ADHS-typischen Psychopathologie. Sie hatten Schwierigkeiten, diese in nosologische Schemata einzuordnen. Innerhalb des Konstrukts der „chronisch-manischen Konzepte“ dieser Epoche sind jedoch deutliche Parallelen zu den heute gängigen Diagnosekriterien der adulten ADHS und ihrer Symptomatik zu erkennen. Viele der damals beschriebenen Patienten würden mit großer Sicherheit heute die Diagnose ADHS erhalten. Diese Feststellung stützt die Hypothese, dass die Diskussionen der vergangenen Zeit als Anhalt dafür dienen können, dass die adulte ADHS ein konstant existentes Krankheitsphänomen darstellt und nicht abwertend als eine „Modediagnose“ abzustempeln ist. Weitere psychiatriehistorische Forschungen, die zunächst den in dieser Arbeit aufgezeigten Berührungspunkten zwischen der deutschen Psychopathielehre zu Beginn des 20. Jahrhunderts und den untersuchten „chronisch-manischen Konzepten“ folgen könnten, scheinen dazu geeignet, die Frage des „Ursprungs“ der adulten ADHS fortführend zu untersuchen.
